# Frameworks for describing health inequalities in observational public health research: a scoping review protocol

**DOI:** 10.1136/bmjopen-2025-106186

**Published:** 2025-09-02

**Authors:** Matylda Buczkowska, Lauren Herlitz, Kate Lewis, Vincent Nguyen, Sorcha Ní Chobhthaigh, Camelia Muldermans, Joseph Lam

**Affiliations:** 1 Institute for Global Health, UCL, London, UK; 2 Social Research Institute IOE - UCL’s Faculty of Education and Society, UCL, London, UK; 3 Institute of Child Health, UCL, London, UK

**Keywords:** Health Equity, PUBLIC HEALTH, EPIDEMIOLOGY, Observational Study, Systematic Review

## Abstract

**Abstract:**

**Introduction:**

Observational studies play a pivotal role in understanding population health trends and informing public health policy. However, many such studies inadequately address dimensions of health inequality, potentially perpetuating existing disparities. There is currently no comprehensive overview of frameworks specifically designed to integrate health-inequality constructs into observational public health research. This protocol outlines the methodology of the scoping review, which aims to identify, describe and critically evaluate existing frameworks that explicitly incorporate health inequalities within observational studies.

**Methods and analysis:**

We will conduct this scoping review in accordance with the Preferred Reporting Items for Systematic Reviews and Meta-Analyses extension for Scoping Reviews guidelines. Six electronic databases (PubMed, Embase, Scopus, Web of Science, Global Health and CINAHL) and eligible grey literature sources will be searched using a combination of keywords and subject headings related to health inequalities, observational study design and frameworks. Two independent reviewers will perform title/abstract screening and full-text eligibility assessment using Rayyan, while discrepancies will be resolved by consultation with a third reviewer. Findings will be synthesised narratively.

**Ethics and dissemination:**

As this study involves analysis of publicly available literature, formal ethical approval is not required. Results will be disseminated through publication in a peer-reviewed journal, presentations at relevant conferences and communication with key stakeholders in public health and equity research. The results will also be shared directly with charities and local organisations which focus on addressing health inequalities. By providing a comprehensive map of existing frameworks, this review will inform researchers on best practices for embedding health-inequality considerations in observational studies and support the development of more equitable research methodologies.

**Strengths and limitations of this study**

- The protocol follows Preferred Reporting Items for Systematic Reviews and Meta-Analyses extension for Scoping Reviews guidelines, ensuring a transparent, reproducible scoping‐review methodology.
- The literature search strategy is comprehensive across multiple electronic databases and includes multiple grey literature data sources, which broadens the scope beyond peer‐reviewed articles to capture non‐indexed frameworks.
- Dual independent screening and data extraction (title/abstract and full‐text stages) with a third reviewer for conflict resolution reduces the risk of selection bias.
- Restricting the search strategy to English‐language sources may result in missing relevant frameworks published in other languages.

## Introduction

 Observational studies are one of the key tools to answer public health research questions. They are particularly useful for studying complex systems and monitoring their impact on human health.^1 2^ Reducing health inequalities is a key priority for policy-makers and researchers within public health research worldwide.^3^,^5^

Growing interest in monitoring population health and its complex determinants has led to an increase in observational studies that use large-scale datasets, which integrate administrative data such as hospital records and education data with biomedical data (including biobank and clinical trial data), and socioeconomic data (including surveys and financial/business data) from broader sources.^6^ Large integrated data integrate administrative datasets such as hospital records and education data with biomedical data (including biobank and clinical trial data), and socioeconomic data (including surveys and financial/business data) from broader sources.^7^ ‘Big Data’ facilitates the collection and analysis of personal characteristics, lifestyle factors, cultural and environmental conditions, living and working conditions and health history.^8^ Despite their potential for exploring differential outcomes and contextual associations, many observational studies fail to acknowledge, critically explore and contextualise health inequalities within populations.^9^

This can be attributed, in part, to limitations in data collection and categorisation practices, which may underrepresent certain groups, such as homeless populations, non-heterosexual families and ethnic minorities. The absence of diverse data has been linked to algorithmic bias, where models trained on homogeneous populations may inaccurately represent what is typical, leading to misclassification or exclusion of under-represented groups.^10^ For example, large-scale cohort studies like the Avon Longitudinal Study of Parents and Children have historically lacked adequate representation of diverse family structures, limiting their ability to capture the experiences and health outcomes of these populations.^11^ This lack of representativeness can skew research findings and policy decisions.^10^ This is further complicated by the uneven power dynamics embedded in how data are generated, framed and interpreted (often referred to as the positionality of both the data and their producers) which can shape what questions are asked, whose experiences are represented and how findings are applied.^10 12^ A lot of observational data also suffers from missing data issues^13^ or might not be collected with specific research objectives in mind, which may introduce limitations related to study design. While causal inference can sometimes be achieved through appropriate methods, the absence of purpose-specific data collection may limit control over confounding factors and variable selection. This disconnect between data availability and conceptual clarity can result in simplistic conclusions that fail to address the structural roots of inequality.

Without explicit focus on health inequalities’ dimensions, observational studies risk reinforcing existing disparities rather than mitigating them. Reporting guidelines designed specifically for observational studies within epidemiological research, such as Strengthening the Reporting of Observational Studies in Epidemiology,^14^ have sought to improve data reporting but do not place significant focus on health inequalities.^15^ The Preferred Reporting Items for Systematic Reviews and Meta-Analyses (PRISMA)-Equity extension provides guidelines for reporting systematic reviews with a focus on equity.^16^ However, it was not specifically developed for public health research. Pedrana *et al*^17^ conducted a scoping review of national monitoring frameworks for social determinants of health, identifying the range and types of indicators used across countries. While their work offers important insights into how social determinants of health are tracked at the national level, it focuses primarily on measurement and monitoring tools, rather than on frameworks that conceptually guide the design or analysis of observational studies in public health research. Similarly, Moorthie *et al*^9^ reviewed approaches to improving the quality of data used to monitor health inequalities, particularly in terms of completeness, standardisation and data governance. However, their focus was on data practices only within healthcare settings. Moreover, a recent study^18^ examining how dimensions of health equity impacts are conceptualised in systematic reviews of public health interventions found that PROGRESS-Plus was the only established framework for this purpose. Despite this, the study’s authors noted that even PROGRESS-Plus does not fully facilitate critical analysis of complex systems and mechanisms.

This lack of clarity is further exacerbated by inconsistent use of terminology such as ‘health inequality’, ‘health inequity’ and ‘health justice’, which, while related, carry distinct normative and conceptual meanings. While health inequalities describe measurable differences in health outcomes between population groups, health inequities refer specifically to those differences that are avoidable, unjust and rooted in structural or social disadvantage.^19 20^ Health equity means that everyone has a fair and just opportunity to be as healthy as possible.^21^ Distinguishing and hence improving definitional clarity is an important step towards enhancing the quality and equity focus of observational public health research.^22^

Additionally, although the concept of social determinants of health is widely used in public health research, there is increasing recognition that such framing can understate the systems of power which produce these conditions. In response, there is growing advocacy for expanding the focus towards structural determinants of health, which highlight and capture how historical, political and economic factors generate and drive health inequalities.^23^,^25^ This perspective emphasises the need for frameworks that not only describe inequality, but also critically examine its root causes.

In this study, a scoping review of existing frameworks which incorporate health inequalities in observational studies will be conducted to improve the quality of public health research on underserved populations. Findings from this review could guide critical thinking on researching health inequalities, drawing attention to its importance and improving research practices.

### Research objectives

The primary objective of this review is to identify and describe the frameworks, which illustrate health inequalities in public health research processes involving observational health data. We will also critically evaluate their comprehensiveness and usability, with particular attention to identifying middle-range theories—theories and frameworks that bridge empirical findings and broader social theory by offering explanations specific enough to be tested, yet abstract enough to apply across multiple contexts. This includes synthesising the various approaches and conceptualisations that underpin how health inequalities, inequities and injustice are understood and operationalised.

## Methods and analysis

This study will be conducted as a scoping review following PRISMA extension for Scoping Reviews guidelines (online supplemental material 1).^26^

### Eligibility criteria

A publication will be included if it:

- Describes or evaluates a framework that illustrates health inequalities including but not limited to inequalities related to healthcare access, service utilisation, disease prevalence, prognosis or treatment outcomes.
- Is a peer-reviewed article or grey literature, such as dissertations, letters, commentaries, reviews, editorials, conference abstracts, governmental files and reports?
- Is explicitly applicable to observational studies (such as administrative data, cohort studies, cross-sectional surveys)?
- Full-text version is available in English.

In cases where a publication includes a conceptual framework focused on health inequalities to inform data collection or an analytical model or approach, we will include it at the abstract screening stage. At full-text screening stage, such papers will only be included if they contain valuable critique or discussion of the framework (eg, in the Discussion section). The original publication of the framework that is referenced will also be sought for screening. If a paper includes an adaptation of an established framework, the adapted framework will be evaluated independently of the referenced framework. Publications that present frameworks focused exclusively on a single social characteristic (eg, gender), condition (eg, substance misuse) or context (eg, environment) will be excluded from the main review. There will be no date restrictions on publication, and the searches will be conducted in English.

### Search strategy

A comprehensive search strategy will be developed to identify relevant literature in major electronic databases, including PubMed, Embase, Scopus, Web of Science, Global Health and CINAHL. Search terms will include variations of keywords related to health inequalities (eg, health inequity, health disparities, health and justice, social determinants of health), observational data and frameworks (online supplemental material 2). Throughout the review, we use a term “framework”, which we define as a structure, overview, outline, system or plan consisting of various descriptive categories, for example concepts, constructs or variables, and the relations between them that are presumed to account for a phenomenon.^27^ However, we recognise that terms such as “guidelines” and “models” are often used interchangeably in the literature. To ensure comprehensive coverage, our search strategy includes these broader terms. While we acknowledge that protected characteristics are central to understanding health inequalities, including an exhaustive set of search terms for all protected variables could make the review unwieldy and impractical. Instead, this review prioritises broader frameworks that capture multiple dimensions of inequality. By first mapping the general landscape of health inequality frameworks, this review will establish a necessary foundation for future research focusing on specific causal pathways. The search strategy will be adapted to each literature database. The search will be supplemented by a manual review of reference lists from included studies to ensure comprehensive coverage.

Grey literature screening will be conducted by reviewing the first 25 pages of Google search results using combinations of key terms related to health inequalities, observational data and frameworks (online supplemental material 2). Key informants in the field will be consulted to identify additional grey literature sources, ensuring the completeness of the included data.

### Screening

Following the literature search, relevant papers will be deduplicated and screened using Rayyan. The study selection will proceed in two stages. The first stage will involve title and abstract screening of all eligible publications by two independent reviewers. In the second stage, full-text screening of all eligible publications will be conducted by two independent reviewers based on the predefined inclusion and exclusion criteria. Any discrepancies in study selection at both title/abstract and full-text screening will be resolved by discussion, with a third reviewer from a wider team consulted where necessary. The literature search, title/abstract screening and full-text review is taking place between April and July 2025.

### Data extraction

Data extraction will be conducted using a custom spreadsheet to capture key study characteristics, including author, year of publication, framework name, theoretical underpinning, core constructs, concepts and features, who the data applies to (eg, has it been created for a specific setting), whether the framework is intended for use for specific aspects of the research process. Two researchers will pilot the data extraction tool on two studies, and discrepancies will be discussed and revisions made where necessary. Data extraction and synthesis are planned for August 2025.

### Quality appraisal

We will critique the strengths and limitations of each model. More specifically, we will primarily focus on assessing applicability (eg, across different analytical levels, populations, settings and observational study designs), usability and practical implementation ease, inclusivity (such as integration of patient and public involvement). Quality of the included frameworks will be assessed using existing ‘Usability Appraisal of the Theories, Models and Frameworks’ criteria, which may be further adapted and tailored to our scoping review as the process progresses.^28^

### Data analysis

The synthesis of findings will be conducted narratively. A priori assumption is to group frameworks based on their theoretical foundations, domains of application and methodological approaches. However, the synthesis factors might be adapted in response to the literature found.

### Patient and public involvement

A participatory focus group of young people will be conducted to critically appraise our chosen methodologies and frameworks, thereby informing and refining our findings.

## Discussion

A key contribution of this review will be its potential to support researchers in navigating complex systems of inequality by providing a comprehensive overview of available conceptual frameworks. This may be particularly valuable in the context of increasingly large and diverse observational datasets, where the risk of reinforcing existing disparities through uncritical analyses remains high.

However, several limitations should be acknowledged. First, the review will be limited to English-language sources, which may result in the exclusion of relevant frameworks developed in non-English-speaking contexts. Second, although the search strategy includes grey literature and reference list screening, some unpublished or informally used frameworks may remain inaccessible. Finally, frameworks emphasising a single axis of inequality (such as gender or disability alone) will be excluded from full synthesis unless they offer broader engagement with health inequality as a concept. This may limit insights into narrowly focused but potentially valuable frameworks.

### Ethics and dissemination

As this study is a scoping review of existing literature, it does not involve primary data collection and does not require ethical approval. These results will then be disseminated to charities and organisations engaged in health‐inequalities research, ensuring that our insights inform practice at community and institutional levels in addition to contributing to academic discourse.

## Supplementary material

10.1136/bmjopen-2025-106186 online supplemental file 1

10.1136/bmjopen-2025-106186 online supplemental file 2
