# Supplementary material for: Frameworks for describing health inequalities in observational public health research: a scoping review protocol
Source: BMJ Open. 2025 Sep 2;15(9):e106186. doi: 10.1136/bmjopen-2025-106186 (PMC12406877; doi:10.1136/bmjopen-2025-106186)
Supplement: online supplemental file 2 [file bmjopen-15-9-s002.docx]

**Supplementary Table 1.** The search strategy terms across all peer-reviewed and grey literature databases utilised in the study.

| **Database** | **Search terms** |
| --- | --- |
| **EMBASE** | (exp health care disparity/ or exp minority health/ or exp health equity/ or exp health inequality/ or exp social stigma/ or exp minority group/ or exp vulnerable population/ or exp social deprivation/ or exp social justice/ or exp social marginalization/ or equit*.ti,ab. or inequit*.ti,ab. or equalit*.ti,ab. or inequalit*.ti,ab. or justice*.ti,ab. or injustice*.ti,ab. or parit*.ti,ab. or gradient*.ti,ab. or gap.ti,ab. or gaps.ti,ab. or disparit*.ti,ab. or underserved.ti,ab. or disadvantage*.ti,ab. or unequal*.ti,ab. or depriv*.ti,ab. or minorit*.ti,ab. or underserv*.ti,ab. or inclus*.ti,ab. or discriminat*.ti,ab. or "protected characteristic*".ti,ab. or vulnerab*.ti,ab. or stigma*.ti,ab. or marginali*.ti,ab.) and (exp epidemiology/ or epidem*.ti,ab. or "public health".ti,ab. or "global health".ti,ab. or "community health".ti,ab. or "population health".ti,ab.) and ("reporting guid*" or "reporting tool*" or "reporting framework*" or "reporting checklist*" or "reporting model*" or "reporting standard*" or "evaluation guid*" or "evaluation tool*" or "evaluation framework*" or "evaluation checklist*" or "evaluation model*" or "evaluation standard*" or "assessment guid*" or "assessment tool*" or "assessment framework*" or "assessment checklist*" or "assessment model*" or "assessment standard*" or "guiding tool*" or "guiding framework*" or "guiding checklist*" or "guiding model*" or "guiding standard*" or "best practice guid*" or "best practice tool*" or "best practice framework*" or "best practice checklist*" or "best practice model*" or "best practice standard*" OR “explanatory framework*” OR “explanatory model*" OR "conceptual framework*" OR "theoretical framework*" OR “conceptual model*” OR “theoretical model*”).ti,ab. and (((record* or registr* or data* or "data base*" or “survey*”) and (computeri?ed or electronic or observational or cohort or "case-control" or "cross-sectional" or longitudinal or retrospective or prospective or admin* or secondary or "routine health" or surveill*)).ti,ab. or (exp medical record/ or exp hospital record/ OR exp Big Data/)) |
| **Global Health** | (exp social stigma/ or equit*.ti,ab. or inequit*.ti,ab. or equalit*.ti,ab. or inequalit*.ti,ab. or justice*.ti,ab. or injustice*.ti,ab. or parit*.ti,ab. or gradient*.ti,ab. or gap.ti,ab. or gaps.ti,ab. or disparit*.ti,ab. or underserved.ti,ab. or disadvantage*.ti,ab. or unequal*.ti,ab. or depriv*.ti,ab. or minorit*.ti,ab. or underserv*.ti,ab. or inclus*.ti,ab. or discriminat*.ti,ab. or "protected characteristic*".ti,ab. or vulnerab*.ti,ab. or stigma*.ti,ab. or marginali*.ti,ab.) and (exp epidemiology/ or epidem*.ti,ab. or "public health".ti,ab. or "global health".ti,ab. or "community health".ti,ab. or "population health".ti,ab.) and ("reporting guid*" or "reporting tool*" or "reporting framework*" or "reporting checklist*" or "reporting model*" or "reporting standard*" or "evaluation guid*" or "evaluation tool*" or "evaluation framework*" or "evaluation checklist*" or "evaluation model*" or "evaluation standard*" or "assessment guid*" or "assessment tool*" or "assessment framework*" or "assessment checklist*" or "assessment model*" or "assessment standard*" or "guiding tool*" or "guiding framework*" or "guiding checklist*" or "guiding model*" or "guiding standard*" or "best practice guid*" or "best practice tool*" or "best practice framework*" or "best practice checklist*" or "best practice model*" or "best practice standard*" OR “explanatory framework” OR “explanatory model" OR "conceptual framework*" OR "theoretical framework*" OR “conceptual model” OR “theoretical model”).ti,ab. and (((record* or registr* or data* or "data base*" OR “survey*”) and (computeri?ed or electronic or observational or cohort or "case-control" or "cross-sectional" or longitudinal or retrospective or prospective or admin* or secondary or "routine health" or surveill*)).ti,ab. or (exp medical record/)) |
| **PubMed** | (equit*[tiab] OR inequit*[tiab] OR equalit*[tiab] OR inequalit*[tiab] OR justice*[tiab] OR injustice*[tiab] OR parit*[tiab] OR gradient*[tiab] OR gap[tiab] OR gaps[tiab] OR disparit*[tiab] OR underserved[tiab] OR disadvantage*[tiab] OR unequal*[tiab] OR depriv*[tiab] OR minorit*[tiab] OR underserv*[tiab] OR inclus*[tiab] OR discriminat*[tiab] OR "protected characteristic*"[tiab] OR vulnerab*[tiab] OR stigma*[tiab] OR marginali*[tiab] OR "Healthcare Disparities"[MeSH] OR "Minority Health"[MeSH] OR "Health Equity"[MeSH] OR "Health Inequities"[MeSH] OR "Social Determinants of Health"[MeSH] OR "Social Stigma"[MeSH] OR "Minority Groups"[MeSH] OR "Vulnerable Populations"[MeSH] OR "Social Deprivation"[MeSH] OR "Social Justice"[MeSH] OR "Social Marginalization"[MeSH]) AND (epidem*[tiab] OR “public health”[tiab] OR “global health”[tiab] OR “community health”[tiab] OR “population health”[tiab] OR "Epidemiology"[MeSH]) AND ("reporting guid*"[tiab] OR "reporting tool*"[tiab] OR "reporting framework*"[tiab] OR "reporting checklist*"[tiab] OR "reporting model*"[tiab] OR "reporting standard*"[tiab] OR "evaluation guid*"[tiab] OR "evaluation tool*"[tiab] OR “evaluation framework*"[tiab] OR "evaluation checklist*"[tiab] OR "evaluation model*"[tiab] OR "evaluation standard*"[tiab] OR "assessment guid*"[tiab] OR "assessment tool*"[tiab] OR "assessment framework*"[tiab] OR "assessment checklist*"[tiab] OR "assessment model*"[tiab] OR "assessment standard*"[tiab] OR "guiding tool*"[tiab] OR "guiding framework*"[tiab] OR "guiding checklist*"[tiab] OR "guiding model*"[tiab] OR "guiding standard*"[tiab] OR “best practice guid*”[tiab] OR “best practice tool*”[tiab] OR “best practice framework*”[tiab] OR “best practice checklist*”[tiab] OR “best practice model*”[tiab] OR “best practice standard*”[tiab] OR “explanatory framework*”[tiab] OR “explanatory model*”[tiab] OR "conceptual framework*"[tiab] OR "theoretical framework*"[tiab] OR “conceptual model*”[tiab] OR “theoretical model*”[tiab]) AND (((record*[tiab] OR registr*[tiab] OR data*[tiab] OR "data base*"[tiab] OR “survey*”[tiab]) AND (computeri?ed[tiab] OR electronic[tiab] OR observational[tiab] OR cohort[tiab] OR "case-control"[tiab] OR "cross-sectional"[tiab] OR longitudinal[tiab] OR retrospective[tiab] OR prospective[tiab] OR admin*[tiab] OR secondary[tiab] OR "routine health"[tiab] OR surveill*[tiab])) OR ("Medical Records"[MeSH] OR "Hospital Records"[MeSH] OR "Epidemiologic Studies"[MeSH] OR “Big Data”[MeSH])) |
| **SCOPUS** | (TITLE-ABS-KEY(equit* OR inequit* OR equalit* OR inequalit* OR justice* OR injustice* OR parit* OR gradient* OR gap OR gaps OR disparit* OR underserved OR disadvantage* OR unequal* OR depriv* OR minorit* OR underserv* OR inclus* OR discriminat* OR "protected characteristic*" OR vulnerab* OR stigma* OR marginali* OR "social determinants of health" OR "health disparities" OR "minority health" OR "health equity" OR "health inequities" OR "social stigma" OR "minority groups" OR "vulnerable populations" OR "social deprivation" OR "social justice" OR "social marginalization")) AND (TITLE-ABS-KEY(epidem* OR "public health" OR "global health" OR "community health" OR "population health")) AND (TITLE-ABS-KEY("reporting guid*" OR "reporting tool*" OR "reporting framework*" OR "reporting checklist*" OR "reporting model*" OR "reporting standard*" OR "evaluation guid*" OR "evaluation tool*" OR "evaluation framework*" OR "evaluation checklist*" OR "evaluation model*" OR "evaluation standard*" OR "assessment guid*" OR "assessment tool*" OR "assessment framework*" OR "assessment checklist*" OR "assessment model*" OR "assessment standard*" OR "guiding tool*" OR "guiding framework*" OR "guiding checklist*" OR "guiding model*" OR "guiding standard*" OR “best practice guid*” OR “best practice tool*” OR “best practice framework*” OR “best practice checklist*” OR “best practice model*” OR “best practice standard*” OR “explanatory framework” OR “explanatory model" OR "conceptual framework*" OR "theoretical framework*" OR “conceptual model” OR “theoretical model”)) AND (TITLE-ABS-KEY((record* OR registr* OR data* OR "data base*" OR “survey*”) AND (computeri?ed OR electronic OR observational OR cohort OR "case-control" OR "cross-sectional" OR longitudinal OR retrospective OR prospective OR admin* OR secondary OR "routine health" OR surveill*))) |
| **Web of Science** | TS=((equit* OR inequit* OR equalit* OR inequalit* OR justice* OR injustice* OR parit* OR gradient* OR gap OR gaps OR disparit* OR underserved OR disadvantage* OR unequal* OR depriv* OR minorit* OR underserv* OR inclus* OR discriminat* OR "protected characteristic*" OR vulnerab* OR stigma* OR marginali* OR "social determinants of health" OR "health disparities" OR "minority health" OR "health equity" OR "health inequities" OR "social stigma" OR "minority groups" OR "vulnerable populations" OR "social deprivation" OR "social justice" OR "social marginalization") AND (epidem* OR "public health" OR "global health" OR "community health" OR "population health") AND ("reporting guid*" OR "reporting tool*" OR "reporting framework*" OR "reporting checklist*" OR "reporting model*" OR "reporting standard*" OR "evaluation guid*" OR "evaluation tool*" OR "evaluation framework*" OR "evaluation checklist*" OR "evaluation model*" OR "evaluation standard*" OR "assessment guid*" OR "assessment tool*" OR "assessment framework*" OR "assessment checklist*" OR "assessment model*" OR "assessment standard*" OR "guiding tool*" OR "guiding framework*" OR "guiding checklist*" OR "guiding model*" OR "guiding standard*" OR "best practice guid*" OR "best practice tool*" OR "best practice framework*" OR "best practice checklist*" OR "best practice model*" OR "best practice standard*" OR “explanatory framework” OR “explanatory model" OR "conceptual framework*" OR "theoretical framework*" OR “conceptual model” OR “theoretical model”) AND ((record* OR registr* OR data* OR "data base*" OR “survey*”) AND (computeri?ed OR electronic OR observational OR cohort OR "case-control" OR "cross-sectional" OR longitudinal OR retrospective OR prospective OR admin* OR secondary OR "routine health" OR surveill*))) |
| **CINAHL** | ((MH "Healthcare Disparities" OR MH "Minority Health" OR MH "Health Equity" OR MH "Health Inequities" OR MH "Social Determinants of Health" OR MH "Social Stigma" OR MH "Minority Groups" OR MH "Vulnerable Populations" OR MH "Social Deprivation" OR MH "Social Justice" OR MH "Social Marginalization" OR TI (equit* OR inequit* OR equalit* OR inequalit* OR justice* OR injustice* OR parit* OR gradient* OR gap OR gaps OR disparit* OR underserved OR disadvantage* OR unequal* OR depriv* OR minorit* OR underserv* OR inclus* OR discriminat* OR "protected characteristic*" OR vulnerab* OR stigma* OR marginali*) OR AB (equit* OR inequit* OR equalit* OR inequalit* OR justice* OR injustice* OR parit* OR gradient* OR gap OR gaps OR disparit* OR underserved OR disadvantage* OR unequal* OR depriv* OR minorit* OR underserv* OR inclus* OR discriminat* OR "protected characteristic*" OR vulnerab* OR stigma* OR marginali*)) AND ((MH "Epidemiology") OR TI(epidem* OR "public health" OR "global health" OR "community health" OR "population health") OR AB (epidem* OR "public health" OR "global health" OR "community health" OR "population health")) AND (TI ("reporting guid*" OR "reporting tool*" OR "reporting framework*" OR "reporting checklist*" OR "reporting model*" OR "reporting standard*" OR "evaluation guid*" OR "evaluation tool*" OR "evaluation framework*" OR "evaluation checklist*" OR "evaluation model*" OR "evaluation standard*" OR "assessment guid*" OR "assessment tool*" OR "assessment framework*" OR "assessment checklist*" OR "assessment model*" OR "assessment standard*" OR "guiding tool*" OR "guiding framework*" OR "guiding checklist*" OR "guiding model*" OR "guiding standard*" OR "best practice guid*" OR "best practice tool*" OR "best practice framework*" OR "best practice checklist*" OR "best practice model*" OR "best practice standard*" OR “explanatory framework” OR “explanatory model" OR "conceptual framework*" OR "theoretical framework*" OR “conceptual model” OR “theoretical model”) OR AB ("reporting guid*" OR "reporting tool*" OR "reporting framework*" OR "reporting checklist*" OR "reporting model*" OR "reporting standard*" OR "evaluation guid*" OR "evaluation tool*" OR "evaluation framework*" OR "evaluation checklist*" OR "evaluation model*" OR "evaluation standard*" OR "assessment guid*" OR "assessment tool*" OR "assessment framework*" OR "assessment checklist*" OR "assessment model*" OR "assessment standard*" OR "guiding tool*" OR "guiding framework*" OR "guiding checklist*" OR "guiding model*" OR "guiding standard*" OR "best practice guid*" OR "best practice tool*" OR "best practice framework*" OR "best practice checklist*" OR "best practice model*" OR "best practice standard*" OR “explanatory framework” OR “explanatory model" OR "conceptual framework*" OR "theoretical framework*" OR “conceptual model” OR “theoretical model”)) AND (((TI (record* OR registr* OR data* OR "data base*" OR “survey*”) AND TI (computeri?ed OR electronic OR observational OR cohort OR "case-control" OR "cross-sectional" OR longitudinal OR retrospective OR prospective OR admin* OR secondary OR "routine health" OR surveill*)) OR (AB (record* OR registr* OR data* OR "data base*" OR “survey*”) AND AB (computeri?ed OR electronic OR observational OR cohort OR "case-control" OR "cross-sectional" OR longitudinal OR retrospective OR prospective OR admin* OR secondary OR "routine health" OR surveill*)) OR (MH "Medical Records" OR MH "Hospital Records" OR MH "Epidemiologic Studies" OR MH “Big Data”)) |
| **Grey literature (first 25 pages of Google results searched)** | “health" OR “global health” OR “public health” OR “healthcare” OR “community health” OR “population health” AND “equity” OR “inequity” OR “equality” OR "inequality" OR "justice" OR “injustice” AND "framework" OR "tool" OR “model” OR “approach” OR “strategy” OR “dashboard” OR “assessment” OR “evaluation” OR “checklist” AND “data” |
