# Supplementary material for: Frameworks for describing health inequalities in observational public health research: a scoping review protocol
Source: BMJ Open. 2025 Sep 2;15(9):e106186. doi: 10.1136/bmjopen-2025-106186 (PMC12406877; doi:10.1136/bmjopen-2025-106186)
Supplement: Supplementary file 3 [file bmjopen-2025-106186.reviewer_comments.pdf]

PEER REVIEW HISTORY

BMJ Open publishes all reviews undertaken for accepted manuscripts. Reviewers are asked to complete a checklist review form and are provided with free text boxes to elaborate on their assessment. These free text comments are reproduced below.

ARTICLE DETAILS

Title (Provisional)

Frameworks for describing health inequalities in observational public health research: A scoping review protocol

Authors

Buczkowska, Matylda; Herlitz, Lauren; Lewis, Kate; Nguyen, Vincent; Ni Chobhthaigh, Sorcha; Muldermans, Camelia; Lam, Joseph

---

VERSION 1 - REVIEW

**Reviewer**	**1**
**Name**	**Marten, Meredith**
**Affiliation**	**University of West Florida**
**Date**	**16-Jul-2025**
**COI**	**None**

This research protocol outlines the researchers' plans for conducting a scoping review of frameworks used across public health research for incorporating data to better address health equity (and its multidimensionality). This is an important project for which the authors make a strong case, and I am excited to read their findings, which I believe will help guide other public health researchers in their efforts to better address health equity.

This protocol is clearly written, and the authors present a compelling background arguing for why this work is needed and will be broadly useful. I have only a handful of comments, and support this work to be published with only a few, very minor, points to address (and consider).

A bit unclear re: the relationship between observational studies and 'Big Data' – that observational studies are increasingly taking the form of 'big data'? Perhaps just phrasing it as "large-scale observational studies, colloquially referred to as 'Big Data'" or something? Or maybe just deleting 'in parallel'?

Great framing of the importance of research (p. 3, 3rd para) – really interesting to consider algorithmic bias and modeling, and the historic biases that may impact/reify categories over

time. So important to note that the biases mentioned here can themselves be considered structural roots of inequality, “reinforcing existing disparities…” –

Also a bit unclear why the participatory focus group (p. 8, line 238) is specifically young people?

Finally, just a note that some folks in anthropology and social/critical epidemiology are increasingly using the social and structural determinants of health, or the social determination of health, or the structural determinants of the social determinants to better focus on the systems/relations of power that undergird the SDOH (eg. the structural competency working group – Seth Holmes, Metzl and Hansen; and some Latin American critical epidemiologists- Jaime Breihl, etc. for some of this excellent work)

This is a really fascinating project that I’m excited to read about and look forward to the authors’ findings. Thanks to the authors for their important work!

**Reviewer**	**2**
**Name**	**Raeesi, Ahmad**
**Affiliation**	**Mashhad University of Medical Sciences**
**Date**	**17-Jul-2025**
**COI**	**None**

Thank you for the opportunity to review your manuscript.

VERSION 1 - AUTHOR RESPONSE

Reviewer: 1
Dr. Meredith Marten, University of West Florida

Comment 2.1: This research protocol outlines the researchers’ plans for conducting a scoping review of frameworks used across public health research for incorporating data to better address health equity (and its multidimensionality). This is an important project for which the authors make a strong case, and I am excited to read their findings, which I believe will help guide other public health researchers in their efforts to better address health equity. This protocol is clearly written, and the authors present a compelling background arguing for why this work is needed and will be broadly useful. I have only a handful of comments, and support this work to be published with only a few, very minor, points to address (and consider).

Response 2.1: Thank you for your positive feedback on the protocol and appreciating the value of the project for the public health researchers.

Comment 2.2: A bit unclear re: the relationship between observational studies and ‘Big Data’ – that observational studies are increasingly taking the form of ‘big data’? Perhaps just

phrasing it as “large-scale observational studies, colloquially referred to as ‘Big Data’” or something? Or maybe just deleting ‘in parallel’?

Response 2.2: We agree that the phrasing was ambiguous and have revised the sentence for clarity:

“Growing interest in monitoring population health and its complex determinants has led to an increase in observational studies that utilise large-scale datasets, which integrate administrative data such as hospital records and education data with biomedical data (including biobank and clinical trial data), and socioeconomic data (including surveys and financial/business data) from broader sources (6).” (lines 73-77, page 3).

Comment 2.3: Great framing of the importance of research (p. 3, 3rd para) – really interesting to consider algorithmic bias and modeling, and the historic biases that may impact/reify categories over time. So important to note that the biases mentioned here can themselves be considered structural roots of inequality, “reinforcing existing disparities…” –
Response 2.3: Thank you very much for your positive feedback on the framing in the protocol, and for acknowledging the role of the biases when addressing health inequalities.

Comment 2.4: Also a bit unclear why the participatory focus group (p. 8, line 238) is specifically young people?
Response 2.4: The authors acknowledge that young people are one of the least engaged group in knowledge generation, hence we explicitly wanted to incorporate young people in this piece to work to have expertise and views from that group and capture their views. Additionally, majority of the authors are working within Child Health Informatics Department and are already drawn to research on young people within their work.

Comment 2.5: Finally, just a note that some folks in anthropology and social/critical epidemiology are increasingly using the social and structural determinants of health, or the social determination of health, or the structural determinants of the social determinants to better focus on the systems/relations of power that undergird the SDOH (eg. the structural competency working group – Seth Holmes, Metzl and Hansen; and some Latin American critical epidemiologists- Jaime Breihl, etc. for some of this excellent work)

Response 2.5: Thank you for this insightful comment. We appreciate the reference to critical perspectives that shift focus from individual-level determinants to the broader social, political, and economic systems that shape health. In response, we have revised the introduction to include reference to the concepts of structural determinants and social determination of health, acknowledging the contributions of Holmes, Metzl and Hansen. These perspectives add important nuance to our framing:

“Additionally, although the concept of social determinants of health is widely used in public health research, there is increasing recognition that such framing can understate the systems of power which produce these conditions. In response, there is growing advocacy for expanding the focus toward structural determinants of health, which highlight and capture how historical, political, and economic factors generate and drive health inequalities (23-25). This perspective emphasises the need for frameworks that not

only describe inequality, but also critically examines its root causes.” (lines 131-137, pages 5)

Reviewer: 2
Dr. Ahmad Raeesi, Mashhad University of Medical Sciences

Comment 3.1: Thank you for the opportunity to review your manuscript.

Response 3.1: Thank you for your time to review our manuscript.
